# The Costs of an Outreach Intervention for Low-Income Women With Abnormal Pap Smears

**Published:** 2006-12-15

**Authors:** Todd H Wagner, Linda P Engelstad, Stephen J McPhee, Rena J Pasick

**Affiliations:** VA Health Economics Resource Center. Dr Wagner is also affiliated with the Department of Health Research and Policy, Stanford University, Stanford, Calif; Alameda County Medical Center, Oakland, Calif; University of California at San Francisco, San Francisco, Calif; University of California at San Francisco, San Francisco, Calif

## Abstract

**Introduction:**

Follow-up among women who have had an abnormal Papanicolaou (Pap) smear is often poor in public hospitals that serve women at increased risk for cervical cancer. This randomized controlled trial evaluated and compared the total cost and cost per follow-up of a tailored outreach intervention plus usual care with the total cost and cost per follow-up of usual care alone.

**Methods:**

Women with an abnormal Pap smear (n = 348) receiving care at Alameda County Medical Center (Alameda County, California) were randomized to intervention or usual care. The intervention used trained community health advisors to complement the clinic's protocol for usual care. We assessed the costs of the intervention and the cost per follow-up within 6 months of the abnormal Pap smear test result.

**Results:**

The intervention increased the rate of 6-month follow-up by 29 percentage points, and the incremental cost per follow-up was $959 (2005 dollars). The cost per follow-up varied by the severity of the abnormality. The cost per follow-up for the most severe abnormality (high-grade squamous intraepithelial lesion) was $681, while the cost per follow-up for less severe abnormalities was higher.

**Conclusion:**

In a health care system in which many women fail to get follow-up care for an abnormal Pap smear, outreach workers were more effective than usual care (mail or telephone reminders) at increasing follow-up rates. The results suggest that outreach workers should manage their effort based on the degree of abnormality; most effort should be placed on women with the most severe abnormality (high-grade squamous intraepithelial lesion).

## Introduction

The U.S. Preventive Services Task Force strongly recommends screening for cervical cancer using the Papanicolaou (Pap) test in sexually active women aged 65 years or younger (1). Cost-effectiveness calculations for cervical cancer screening are sensitive to the periodicity with which women are screened and the prevalence or likelihood of cervical cancer in the population (2-4). Many health care organizations recommend screening every two or three years, unless the woman is at increased risk. Efforts to find and to screen high-risk populations are frequently more cost-effective than general population-based screening strategies. A recent study showed that it is more cost-effective to focus on women who are rarely or never screened rather than on women who have had a prior normal Pap smear (5).

Even when women get screened, follow-up is not perfect. Published cost-effectiveness models frequently make assumptions about the rate of follow-up; for instance, Goldie et al assumed a 15% loss to follow-up with each visit (6,7). Unfortunately, loss to follow-up can be much higher in some populations. Past studies have shown that up to 64% of low-income and ethnically diverse women fail to get adequate follow-up after an abnormal Pap smear (8-12). Although there is evidence that reminder and educational interventions are efficacious at improving recommended follow-up compared with usual care (13,14), one study indicates that these interventions are less successful for low-income, ethnically diverse women (10). Therefore, when health care providers screen low-income or ethnically diverse women for cervical cancer and the test result is abnormal, additional efforts to increase follow-up may be warranted.

This randomized controlled trial evaluated and compared the cost and cost per follow-up of a tailored outreach intervention plus usual care with the cost and cost per follow-up of usual care alone. Community health advisors (CHAs) provided the tailored outreach through telephone and in-person contact. In this study, we focused on women with an abnormal Pap smear at a county hospital that serves a low-income, racially and ethnically diverse population.

## Methods

This study used outreach and tailored individual counseling to improve follow-up among high-risk, public hospital patients with an abnormal Pap smear. We report on the efficacy of the study and details of the intervention elsewhere (15). Institutional review boards at the Northern California Cancer Center, University of California at San Francisco, and the Alameda County Medical Center approved the study protocol.

The intervention was conducted at Alameda County Medical Center (ACMC), which is the acute care public hospital in Alameda County, California. Alameda is an urban, industrialized county on the east side of the San Francisco Bay Area. The county has a population of approximately 1.44 million residents, with a large concentration in the city of Oakland. Fifty-nine percent of the county population is nonwhite (16). ACMC has ambulatory care programs and an emergency department that together provide outpatient care to more than 120,000 women annually, many of whom are low income. Of the 4139 medical–surgical hospitals in the United States, ACMC ranked 38th in 2004 (first percentile) in its provision of care to Supplemental Security Income (SSI) and Medicaid beneficiaries through the Medicaid Disproportionate Share Hospital program, which makes payments to hospitals that serve disproportionate numbers of low-income patients with special needs (17).

### Sample 

Women with an abnormal Pap smear recorded in ACMC's laboratory database from September 1, 1999, to August 31, 2001, were eligible to participate in the study. The database was reviewed weekly during the study to identify eligible women. An abnormal Pap smear included atypical squamous cells of undetermined significance (ASCUS), atypical glandular cells of undetermined significance (AGUS), low-grade squamous intraepithelial lesion (LGSIL), and high-grade squamous intraepithelial lesion (HGSIL), according to the Bethesda system (18).

Women were excluded if they were aged 18 or younger or older than 74, did not speak any English or Spanish, lived outside of Alameda County, were in the process of following up their abnormal Pap smear, or were pregnant with an estimated date of delivery later than October 31, 2001 (which would have required outreach after the intervention period). We randomized all eligible women. Because the study was viewed as a quality improvement initiative, consent was not required, and the introduction of selection bias at the point of randomization was thus eliminated. Randomization was stratified by degree of abnormality (ASCUS, AGUS, LGSIL, and HGSIL) to balance the intervention and control group. Analyses were based on intention to treat.

The main outcome was timely follow-up of the abnormal Pap smear. In most cases, this was operationalized as the initiation of follow-up within 6 months of the date of the original abnormal Pap smear. Unless the abnormal result was HGSIL, pregnant women or women who became pregnant before the 6-month follow-up were encouraged to seek follow-up care shortly after delivery. Therefore, timely follow-up for pregnant women was defined as initiation within 3 months after the expected due date.

A total of 515 women with an abnormal Pap test were identified; of these, 348 women were eligible. We randomized 178 women to the intervention group and 170 to the usual care group.

### Intervention 

The intervention took place from January 2000 through January 2001. The three CHAs were women; two were African American and one was Latina and bilingual in English and Spanish. All three had prior community health outreach experience. The CHAs were trained on the study protocol, were monitored by a research coordinator, and were aided by a computerized participant tracking system. The content of the intervention was developed from formative research and closely matched constructs from the Health Belief Model (19) and Social Cognitive Theory (20).

The CHAs were housed in an office at ACMC. This location gave them access to hospital databases and placed them in close proximity to the clinics. The CHAs contacted participants in the intervention group by telephone to administer a brief demographic questionnaire. Following the initial telephone contact, CHAs met with each woman, administered a structured baseline questionnaire, and using an algorithm based on responses to the survey, provided tailored education, counseling, and referrals. The primary objective of the CHAs throughout the intervention was to help the women achieve timely follow-up of an abnormal Pap smear. This assistance typically included reminder telephone calls and help with scheduling appointments. CHAs used hospital records to ascertain whether appointments were kept, missed, cancelled, or rescheduled, and they confirmed this information by contacting the participants. If follow-up was not obtained, the CHAs helped schedule another appointment and investigated whether there were any further barriers to care. More details of the intervention are reported elsewhere (15).

### Usual care

ACMC provided all study participants with usual care that included notification by telephone or mail. The type of follow-up protocol by the clinic nurse or designee depended on the degree of Pap smear abnormality. Women with the most severe abnormality (HGSIL) were advised to have a colposcopy within 2 weeks of the receipt of the test result. Women with AGUS or LGSIL were advised to have a colposcopy within 6 to 8 weeks of the receipt of the test result. Finally, women with ASCUS were advised to have another Pap smear, followed by a colposcopy, depending on the second test result.

### Rescue 

Women in the usual care group who did not have a follow-up appointment after 6 months were assigned to the CHAs for rescue. Women assigned to rescue were treated the same as women in the intervention group. We tracked the time spent on rescue by CHAs and excluded these costs from the cost-effectiveness analysis.

### Cost data 

The CHAs helped develop forms to document each time they talked to a participant or took action on her behalf. The forms tracked time and incidental costs associated with each participant and provided detailed information on the incremental time spent with each participant. (The client contact form used during the intervention is available in the Appendix.) We then computed a cost estimate for the time spent on direct participant contact by using an hourly wage with benefits ($22.28) based on administrative records.

We recorded 426 hours of intervention time (direct participant contact) or approximately 2.4 hours per woman in the intervention group. Study payroll records indicated that the CHAs were paid for 1148 hours. After subtracting the 426 hours, we assumed that the remaining 722 (63%) hours were spent performing intervention-related work that did not include direct participant contact, such as participation in training and meetings. Our expectation had been, however, that CHAs would spend 10% to 15% of their time on intervention-related work. Upon further investigation, the CHAs reported providing help on other projects and studies during slow times. We concluded that the 722 hours included these other projects and studies. The data did not allow us to identify how much time, if any, should be excluded. Therefore, we took a conservative approach and included all 722 hours in the primary analysis.

In addition to the CHAs, the intervention employed a supervisor who met weekly with the CHAs. This person provided training and quality assurance for the CHAs; for example, the supervisor reviewed CHA forms for completeness and accuracy. We calculated a total cost for quality assurance ($35 per hour with benefits) and then distributed this cost uniformly to each study participant. Finally, we tracked office space and supply costs.

Usual care was either a telephone call or a letter; the choice depended on the degree of Pap smear abnormality. We estimated the cost of a telephone call at $3 (labor) and the cost of a letter at $1 (postage and labor). We estimated patient travel costs by using straight-line distance between the patient's ZIP code and ACMC (21). Patient travel costs reflected a combination of travel expenses and travel time. We estimated travel expenses at $0.365 per mile based on federal mileage reimbursement rates (22). We estimated the cost of travel time by assuming 1.5 minutes per mile and $19.04 per hour based on the Bureau of Labor Statistics average blue-collar wage rate for the San Francisco and Oakland areas (23). Travel costs were incurred only for women who had follow-up. All costs were standardized to 2005 dollars using the Bureau of Labor Statistics Price Index for all urban consumers (23). We assessed the association between the number of hours CHAs spent with participants and number of contacts using Pearson's correlation coefficient.

### Cost per follow-up

We calculated the incremental cost per follow-up by using a societal perspective. We included all costs regardless of who incurred the costs during the study (24). In a secondary analysis, we calculated the incremental cost per follow-up by using a payer perspective. We determined the cost of adding the intervention to usual care assuming only the costs that the payer would bear. Extending the analysis to include lifetime costs and benefits was beyond the scope of the study.

The unit cost of the follow-up visit ($195) was calculated as the weighted average of Medicare's payment for colposcopy current procedure terminology (CPT) codes. The ambulatory payment classification (APC) facility payment was included. Colposcopy CPT codes have similar reimbursement rates, with the exception of colposcopy with loop electrode biopsy. Calculating a weighted average corrects for this exception because electrode biopsy is a rare procedure. We used 2004 data from the Department of Veterans Affairs to identify the frequency with which these CPT codes were used (25).

Bootstrapping with 1000 replications enabled us to calculate 95% confidence intervals (CIs) for the incremental cost-effectiveness ratio (26). Bootstrapping also enabled us to calculate nonparametric CIs without making assumptions about data distributions.

We ran two sensitivity analyses to identify whether the results held when input parameters were changed. Because wages in the San Francisco Bay Area are substantially higher than in other urban areas of the United States, we employed a sensitivity analysis using the national average for a customer service representative (27). Second, we assumed that the 426 hours of CHA time represented 85% of their time and the remaining 15% was spent in meetings or in training; this assumption was consistent with a recent mammography study (28).

## Results

### Sample characteristics 

The intervention and control groups were not statistically different in terms of race and ethnicity, primary language, age, pregnancy status, reason for Pap smear, severity of Pap smear abnormality, insurance status, or distance from home to ACMC (Table 1). Approximately half of the sample was African American and between the ages of 18 and 29 years. The majority was not pregnant (83%) and received an abnormal screening on a routine Pap smear (61%). Overall, 20% of the participants had either LGSIL or HGSIL. All of the participants lived within 40 miles of ACMC, except for four participants, all of whom were in the control group. The mean travel distance was 7 miles for the intervention group and 11 miles for the control group, but the median distance was similar (5 miles, intervention; 4 miles, control).

### Cost per woman

CHAs spent an average of 2.39 hours per intervention group participant. After reconciling these data with the administrative records, which allowed us to include indirect time, the mean time spent per woman was 6.38 hours, or approximately $155 (Table 2). The remaining intervention-related costs included CHA quality assurance ($21 per intervention participant) and office space and supplies ($31 per intervention participant). The average cost of patient travel was $20 for women in the intervention group. The follow-up visit cost $195 for those who had a follow-up visit. In sum, the cost of the intervention averaged $355 per woman from the societal perspective. The cost of usual care averaged $1. Table 2 also shows that from the payer perspective, the intervention cost $335 for the intervention group and $67 for the control group.

### Cost per follow-up

The incremental cost per follow-up was $959 (95% CI, $787–$1367) from the societal perspective and $926 (95% CI, $754–$1333) from the payer perspective (Table 3).

We calculated the cost per follow-up for women with ASCUS/AGUS, LGSIL, and HGSIL. As shown in Table 3, the costs did not vary much among these three groups, but the effectiveness did. In fact, the intervention improved follow-up for women with the most severe abnormality (HGSIL). There were 15 such women in the intervention group and 14 in the control group. After the intervention, of the women with HGSIL, 87% of those in the intervention group and 43% of those in the control group had documented follow-up.

The sensitivity analysis showed that the results were relatively robust to the wage rate. In areas where CHAs would be paid $17 per hour (compared to $22 in this study), the cost per follow-up would be reduced from $959 to $836. The results were more sensitive to assumptions about the time CHAs spent in meetings or in training (indirect costs). If we assumed that CHAs spent 85% of their time providing services, then the average time a CHA spent with a woman would be 2.8 hours (instead of 6.38 hours), and cost per follow-up would decrease from $959 to $660.

### Staffing

For each woman in the intervention, the average costs were the following: CHA time, $155; quality assurance, $21; office space and supplies, $31. The total cost of the intervention for each woman was $207 and for all 178 women, $36,846. The Figure shows data on the total number of hours spent with participants, the total number of contacts made, and the total number of HGSIL contacts made by the three CHAs each month. The Figure illustrates the close relationship between the total number of contacts and total number of hours (Pearson *r* = 0.54; *P* < .001). Also evident is the wide variability in workload over time.

**Figure:**
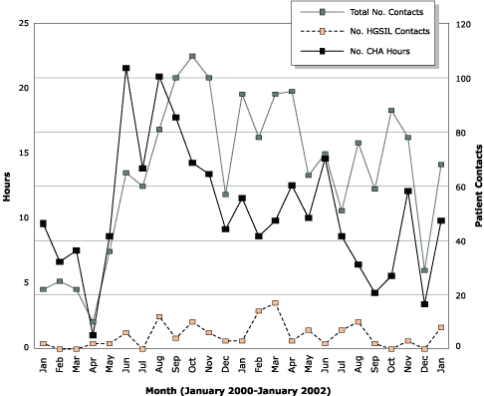
Number of hours spent and number of contacts made per month by three community health advisors (CHAs) in an intervention designed to increase follow-up on abnormal Pap smears, January 2000 through January 2002 (Pearson *r* = 0.54; *P* < .001). Not shown are November and December 1999 in which four contacts were made. HGSIL indicates high-grade squamous intraepithelial lesion.

**Table:** 

## Discussion

The CHAs were effective at increasing follow-up among low-income, racially and ethnically diverse women who have had an abnormal Pap smear. The incremental cost per follow-up was $959 (2005 dollars). The total cost for the outreach program for 178 women, including patient time and follow-up care, was $50,132 more than usual care. Although this program would cost less in other parts of the country where labor is less expensive than in the Bay Area, some health care providers may not be able to afford the program. An alternative would be for a health care provider to use usual care for women with ASCUS and AGUS and to use CHAs for LGSIL, HGSIL, or both. The cost per follow-up was lower (more favorable) for women with HGSIL than for all other women. Targeting the program on more severe abnormalities may make sense because women with more severe abnormalities face the greatest risk of cancer and have the most to gain from obtaining follow-up.

In this study, 38% of the women receiving usual care had follow-up within 6 months after the Pap smear. Because we conducted the study in only one health care institution, we can only speculate on why usual care was less effective than what is often reported in the literature (10,29). In fact, the study site is unique, which may explain the success of the intervention as well as the poor response to usual care. ACMC is a county facility treating a high proportion of uninsured women who face barriers to care and have low health literacy. Many patients at ACMC have limited financial resources and face cultural or language barriers or both. Some have substance-use problems and practice unsafe sexual behaviors and are at higher risk for human papillomavirus and human immunodeficiency virus. The outreach workers were trained to help the women overcome many of the barriers they face. But until more research is conducted in other settings, it is not known whether this program could be transferable to other county hospitals, public health systems, or managed care programs.

We found that CHAs were successful at increasing rates of follow-up in a health care system that uses conventional cytology. Recent clinical trials suggest that the conventional management of cervical cancer screening creates barriers because multiple visits are needed to screen, conduct follow-up, and treat. Single-visit screen-and-treat approaches were more effective than usual care (30,31). In populations in which many barriers inhibit adequate follow-up, screen-and-treat approaches were also cost effective compared with usual care (7). However, these protocols do not appear to change the long-term screening behaviors of most women. Brewster et al found no differences between intervention and control groups in obtaining a Pap smear a year later except for women with HGSIL/AGUS (31). Even for women with HGSIL/AGUS in the intervention group, only 63% had a Pap smear a year later; this percentage leaves room for improvement (31). Consequently, health care systems that adopt screen-and-treat protocols may find value in combining screen-and-treat protocols with educational interventions, such as the use of CHAs.

The primary limitation of this study is that the incremental cost per follow-up is an intermediate outcome and assumes that greater rates of follow-up lead to improved survival and quality of life. Most cost-effectiveness analyses, however, use quality-adjusted life-years (QALYs) for measuring effectiveness (24). Modeling the incremental cost per QALY was beyond the scope of this study.

A second limitation of this study is the larger than expected difference between the time recorded on the CHA logs and the time reported in the CHA payroll records. These two time estimates were not expected to match perfectly because the CHA logs only record time spent on direct contact with participants. The CHA logs do not record time spent in meetings or in training. We expected the administrative records to show that 10% to 15% of the CHAs' time was spent on meetings and training, but we found that 63% of their time was spent on activities that did not involve direct contact with participants. This information suggests that our cost findings are probably high. It also highlights the fact that CHA workload is not uniformly distributed over time (Figure). Health care systems that adopt this type of intervention should pay particular attention to the volume of abnormal Pap smears. Health care systems with electronic databases of laboratory results can identify the number of abnormal Pap smears over time. They can then make an informed decision about the number of full-time equivalent staff needed to meet the workload and how they manage variations in workload. Health care systems could also look into other ways of paying CHAs. However, alternative payment methods could affect incentive structures for CHAs, which could, in turn, affect follow-up rates.

In summary, the use of CHAs to promote follow-up after an abnormal Pap smear is both more costly and more effective than usual care. The incremental cost per follow-up was $959 (2005 dollars), and this amount would likely be smaller in other areas of the country where labor costs are less than they are in the Bay Area. These findings are particularly relevant for public health care systems where low-income and racially and ethnically diverse women seek care.

## Figures and Tables

**Table 1:** Sample Characteristics of Intervention Group (Community Health Advisors Plus Usual Care) and Control Group (Usual Care), Alameda County, California, 2000–2002

Characteristic	Intervention Group, % (n = 178)	Control Group, % (n = 170)	*P* Value^a^
Race			
African American	49.4	49.4	.31
Latina	37.1	32.9	
White	6.7	5.3	
Other	6.7	12.4	
English speaking	65.7	68.2	.62
Age, y			
18-29	46.6	50.0	.63
30-39	21.9	24.7	
40-49	18.5	14.1	
50-74	12.9	11.2	
Pregnant	17.4	15.9	.70
Reason for Pap test			
Routine	64.0	58.2	.53
Diagnostic	33.7	39.4	
Unknown	2.2	2.4	
Result of Pap test			
ASCUS/AGUS	79.2	80.0	.98
LGSIL	12.4	11.8	
HGSIL	8.4	8.2	
Insurance status			
Private	0.6	0	.71
Public non-HMO	13.5	11.8	
Public HMO	16.3	19.4	
Uninsured	59	55.9	
Unknown	10.7	12.9	
Distance from home to hospital, mean miles (SD)	7.0 (8.8)	11.3 (47.5)	.24

Pap indicates Papanicolaou; ASCUS, atypical squamous cells of undetermined significance; AGUS, atypical glandular cells of undetermined significance; LGSIL, low-grade squamous intraepithelial lesion; HGSIL, high-grade squamous intraepithelial lesion; HMO, health maintenance organization.

a *P* value for categorical variables was determined using Pearson's chi-square test, and for continuous variables, *t* test.

**Table 2:** Comparison of Average Payer and Societal Costs per Woman (U.S. Dollars in 2005) in Intervention Group (Community Health Advisors Plus Usual Care) and in Control Group (Usual Care), Alameda County, California, 2000–2002^a^

Cost	Intervention Group Mean (SD) (n = 178)	Usual Care Mean (SD) (n = 170)
Payer costs		
Outreach worker costs	155 (115)	0 (0)
Travel costs at .365 per mile	4 (7)	0 (0)
Office space and supplies	31 (0)	0 (0)
Outreach worker quality assurance	21 (0)	0 (0)
Usual care	1 (1)	1 (1)
Follow-up visit	123 (99)	65 (95)
Patient travel costs for follow-up visit	20 (21)	11 (20)
Total average unit cost from societal perspective^b^	355 (182)	77 (111)
Total average unit cost from payer perspective^c^	335 (169)	67 (95)

a A standard deviation of 0 indicates a fixed cost.

b Societal perspective includes all costs regardless of who would bear them.

c Payer perspective includes only the costs that the payer would bear.

**Table 3:** Incremental Societal and Payer Costs (U.S. Dollars in 2005) per Follow-up for Intervention Group (CHA Plus Usual Care) and Control Group (Usual Care), Alameda County, California

Perspective	Cost, $	Incremental Cost, $	Rate of 6-Month Follow Up	Increase in Rate of 6-Month Follow Up	**Incremental Cost for Follow-Up, $ (95% CI)**
**Societal perspective^a^**					
Control	77	—	0.32	—	—
Intervention	355	278	0.61	0.29	959 (787-1367)
**Payer perspective^b^**					
Control	67	—	0.32	—	
Intervention	335	268	0.61	0.29	926 (754-1333)
**Societal perspective by result of Pap test**					
ASCUS/AGUS					
Control	75	—	0.32	—	—
Intervention	347	272	0.57	0.25	1090 (813-1658)
LGSIL					
Control	74	—	0.30	—	—
Intervention	374	300	0.64	0.34	882 (579-4584)
HGSIL					
Control	105	—	0.43	—	—
Intervention	405	300	0.87	0.44	681 (486-1989)

CI indicates confidence interval; Pap, Papanicolaou; ASCUS, atypical squamous cells of undetermined significance; AGUS, atypical glandular cells of undetermined significance; LGSIL, low-grade squamous intraepithelial lesion; HGSIL, high-grade squamous intraepithelial lesion.

a Societal perspective includes all costs regardless who would bear them.

b Payer perspective includes only the costs that the payer would bear.

## Appendix

The client contact form used in this study is available for download as a Microsoft Word document. You must have Microsoft Word to use this file.

**Download the Client Contact Form** (DOC 103k)
