# Supplementary material for: The Costs of an Outreach Intervention for Low-Income Women With Abnormal Pap Smears
Source: Prev Chronic Dis. 2006 Dec 15;4(1):A11. (PMC1832136)
Supplement: Supplementary file 1 [file 06_0058_02.doc]

#
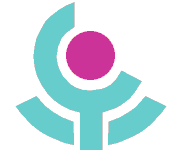
`

**CHA Name:**

**Client Contact Form**

**Pathfinders, Project #2**

**NOTE:** Complete every time you talk to a woman or talk to someone on her behalf.

Contact Information

**Client's Name: _______________________ Phone#________________ ID#: ________**

| **Attempts to Contact by Phone (Date, Time, Call Result) Example #1: 1/2/00, 8:00pm, Message left Example #2: 1/2/00, 8:00pm, No Answer NOTE: Home visit if "no contact" after a minimum of 5 call attempts.** | | |
| --- | --- | --- |
| 1) | | 6) |
| 2) | | 7) |
| 3) | | 8) |
| 4) | | 9) |
| 5) | | 10) |
| **Reason for Contact** | | |
| Administer pre-survey | Remind her of appointment | |
| Administer survey | Check if she kept appointment | |
| Provide consultation/referral information | Other(specify): | |
| **Contact Details**  Contact Date: Contact Time:  Contact To / From (circle one):  Contact Type: Phone **or** In Person (specify location):    **Total Time with Client*:** **Travel Time*:** **Expenses:**   | Hours | Minutes |  | Travel Only | Hours | Minutes |  | Mileage | Parking | | --- | --- | --- | --- | --- | --- | --- | --- | --- | |  |  |  | No Contact |  |  |  |  |  | | | |

| **Appointment Information**  Contact Outcome | |
| --- | --- |
| Appointment date: Time: Clinic: | |
| Appointment kept?: Yes No, why?  Cancelled Rescheduled appt, date/time | |
| Date to Date to  give reminder call**: check if appointment was kept**: | |
| **Consultation** | |
| **A.** Consumer skills | Coping Skills: |
| (blue/green/pink/yellow) | **E.** Distancing |
| **D**. Appointment Magnet | **F.** Seeking Social Support |
| Abnormal Pap Education | **G.** Escape Avoidance |
| Other (specify): | **H.** Problem Solving |
| **Referrals** | |
| **B.** Transportation | **K.** Substance abuse |
| AC Transit Voucher | **L.** Domestic violence |
| **C.** Child care | **M.** Sexual abuse |
| **I.** Mental Health | **V.** HIV/AIDS |
| **J.** Alcohol abuse | Other (specify): |
| **Outreach Plan** | |
|  | |
|  | |
|  | |

* See Reverse

**Enter Dates in calender book

| *** Prolonged Contact or Travel Times**  **Please explain any unusually long contact times due to interruptions, delays, long waits or other reasons.** |
| --- |
|  |
|  |
|  |
|  |
|  |
|  |
| Contact Notes & Comments |
|  |
|  |
|  |
| **General Comments** |
|  |
|  |
|  |
|  |
|  |
|  |
|  |
|  |
|  |
|  |
|  |
|  |
|  |
|  |
|  |
|  |
|  |
|  |
|  |

* See Reverse
